# Ischemic heart disease risk factors in lead exposed workers: research study

**DOI:** 10.1186/1745-6673-8-11

**Published:** 2013-04-22

**Authors:** Masoumeh Ghiasvand, Kamran Aghakhani, Ahmad Salimi, Ranjit Kumar

**Affiliations:** 1 Occupational Medicine Specialist, Faculty of Medicine, Iran University of Medical Sciences, Tehran, Iran; 2 Forensic Medicine Specialists, Faculty of Medicine, Iran University of Medical Sciences, Tehran, Iran; 3 Consultant Aerospace Medicine, Centre for Advanced Research and Development, Bangalore, India

**Keywords:** Lead, Hypertension, Hyperlipidemia, Hyperglycemia, Ischemic heart disease

## Abstract

**Background:**

Review of other epidemiological studies reveal inconsistent results of relationships between high blood lead level and risk of hypertension, hyperlipidemia and hyperglycemia. In this study we wanted to find if there is a relationship between blood lead level and these ischemic heart disease risk factors.

**Methods:**

This cross-sectional study was conducted in a battery recycling plant, and 497 male workers with the mean age of 41.7 (±6.50) years were recruited from all over the plant (those from the products and maintenance sections were classed as “high lead exposed group” and those from amongst the office, laboratory, security services and food services sections as “low lead exposed group”). Personal information such as demographics and work history was obtained through a questionnaire.

Mean (±Standard deviation) for quantitative variables, Frequency (Percent) for qualitative variables, and Odd’s ratio (OR) with 95% confidence interval (95% CI) for estimating the effect of blood lead level on lipid profile[triglyceride (TG), cholesterol(CHOL), low density lipoprotein – Cholesterol(LDL-C),high density lipoprotein –Cholesterol(HDL-C)], hypertension(HTN) and fasting blood sugar (FBS) level. Logistic regression modeling was used for multivariate analysis and adjusting the effect of different variables (age, body mass index(BMI), eating habits, cigarette smoking).

**Results:**

The mean Blood Lead Level (BLL) was >40 μg/dl in 281 (56.6%) subjects, ≤40 μg in 216 (43.4%) subjects and the mean BLL was 43.3 μg/dl (n = 497). The mean job experience involving lead exposure was 13 years. There was no significant correlation between BLL and FBS (p = 0.68), between BLL and TG (P = 0.32), between BLL and HDL-C (p = 0.49), between BLL and LDL-C (p = 0.17), between BLL and CHOL(p = 0.96), between BLL and systolic blood pressure (p = 0.12). The adjusted Odd’s ratio for the effect of BLL >40.0 μg/dl on diastolic blood pressure was1.03 (95% CI: 1.01–1.05) with p = 0.05.

**Conclusion:**

This study showed an association of high BLL with diastolic blood pressure but not with TG, FBS, and HDL-C, LDL-C and CHOL . This result persisted even after adjustment was made for age, BMI and job experience, smoking and eating habits. Attention to health-protective policies, individual behavioral changes and regular periodic medical examination with focus on diastolic blood pressure in lead exposed workers is likely to decrease the public health burden of ischemic heart disease.

## Background

The major risk factors in Ischemic Heart Disease (IHD) are physical inactivity, high serum lipids/cholesterol, hypertension, family history of IHD, obesity, smoking and gender. Environmental pollution and occupation exposure to some chemicals and metals (lead, arsenic, mercury and cobalt) have also been known to contribute to cause or to exacerbate IHD. Lead is a heavy metal and is widely used in various industrial processes. One of the major uses of lead is in batteries manufacturing/recycling units for automobile industry. Workers in a battery manufacturing plant are easily exposed to lead. Exposure occurs through inhalation, ingestion or occasionally by direct skin contact. It is a toxic substance and affects every one of the body’s organ systems. It adversely affects not only the nervous system, but also the cardiovascular system, the skeleton, kidneys, haemopoetic system, and reproductive systems [1]. Hearing loss and tooth decay have been linked to lead exposure [2,3].

Lead is also considered as one of the contributing factors to hypertension [4]. However, recent study has revealed the effect of lead on arterial blood pressure at levels not causing clinical symptoms of renal impairment [5]. Cumulative low-level lead exposures are associated with elevated blood pressure and thereby may increase the risk of atherosclerotic cardiovascular disease [6]. Many in vitro and in vivo studies have shown the possible mechanisms by which lead exposure can raise arterial pressure. These are: by promoting oxidative stress and inflammation, disturbing NO (Nitrous Oxide) signaling pathways, altering major vasoregulatoy systems, damaging endothelial lining, promoting vascular smooth muscle cells(VSMC) proliferation and transformation, and inhibiting fibrinolysis, [7-14]. The effects of lead poisoning in diabetes subjects have been recognized [15-17]. Epidemiological and experimental studies in animals have shown lead toxic effects at high levels of exposure and it is possible that high blood concentration of lead may contribute progression of diabetic complications in diabetic patients [16,18,19].

The aim of the present epidemiological study was to investigate whether blood lead level is associated with lipid, fasting blood sugar and hypertension disturbances.

## Material and methods

A cross-sectional study was conducted among 497 male workers of a recycling battery manufacturing plant. The questions covered various aspects of working conditions, smoking habits, and family history of hypertension, diabetes, hyperlipidemia, and hypothyroidism. Hypertension was defined as having a systolic blood pressure of 140 mmHg or more, or a diastolic blood pressure of 90 mmHg or more and or answering “yes” about being on antihypertensive medication. Body weight and height were measured in light indoor clothing and recorded to the nearest kilogram. and nearest centimeter respectively. Body mass index (BMI) was calculated and those with a BMI of 30 or more were classified as obese. 233 workers who had direct lead exposure from the products and maintenance parts were classed as “high lead exposed group” and 264 workers was selected from amongst the office workers, lab technicians, security services and food services of the factory as “reference group”. These workers were not exposed to lead.

After an overnight fasting of 12 hours, blood samples were collected between 08:00–10:00 hrs for BLL and other biochemical analysis. Total cholesterol, triglycerides and HDL-cholesterol concentration were measured. Aliquots of blood samples were separated for lead analysis which was measured by atomic absorption spectrophotometer and the remaining blood samples were centrifuged to separate plasma and red blood cells. Serum triglycerides, cholesterol and FBS levels were measured by enzymatic assays, HDL and LDL by direct immuno turbidometry assays (Pars-Azmun, Tehran, Iran). Triglyceride value >200 mg/dl, HDL-cholesterol <45 mg/dl, Cholesterol > 200 mg/dl, LDL > 130 mg/dl were defined as lipid disturbances.

The study protocol was approved by the Ethical Committee of the Occupational Medicine Department Tehran University of Medical Sciences, Tehran, Iran.

The SPSS software version 16 was used for statistical analysis. Mean (±Standard deviation),for quantitative variables, Frequency (Percent) for qualitative variables, and Odd’s ratio (OR), and 95% confidence interval (95% CI) was used for estimating the effect of blood lead level on lipid profile, hypertension and high blood glucose levels. Logistic regression modeling was used for multivariable analysis and adjusting the effect of different variables (age, BMI, eating habits, cigarette smoking).

## Results

The total of 497 battery recycling male workers were consisting of 281 (56.6%) with BLL >40 μg/dl and 216 (43.4%) with BLL ≤40 μg/dl. The mean age of the workers was 41.7 ± 6.5 years. The mean duration of job experience of the workers was 12.90 ± 7.00 years. Mean blood lead level was 43.31 (7–94) μg/dl ± 17.95.

Table 1 shows the result of mean of the cardiovascular variables in 2 groups (group1: BLL > 40 μg/dl and group2: BLL ≤ 40 μg/dl).

**Table 1:** Mean of variables in 2 groups(group1: BLL > 40 μg/dl and group2: BLL ≤ 40 μg/dl)

**Variables**	**Group1**	**Group2**
Fasting Blood Sugar	86.75	91.43
Triglyceride	155.90	172.95
Cholesterol	191.60	190.70
LDL-C	115.94	119.43
HDL-C	40.90	41.00
SystolicBlood Pressure	119.68	118.40
Diastolic Blood Pressure	73.04	72.68
Body Mass Index	26.43	26.20

LDL-C: Low Density Lipoprotein-Cholesterol, HDL-C: High Density Lipoprotein-Cholesterol.

Table 2 shows the result of mean of the cardiovascular variables in all workers.

**Table 2:** Mean (±SD) of variables in all workers

**Variables**	**Mean**	**Standard deviation(±SD)**
Fasting Blood Sugar mg/dl	89.22	± 25.25
Triglyceride mg/dl	164.85	± 109.92
Cholesterol mg/dl	191.12	± 55.68
LDL-C mg/dl	117.80	± 39.40
HDL-C mg/dl	40.96	± 11.93
Systolic Blood Pressure mmHg	119.00	± 10.80
Diastolic Blood Pressure mmHg	72.85	± 7.77
Blood Lead Level μg/dl	43.31	± 17.95
Body Mass Index	26.31	± 3.56

LDL-C: Low Density Lipoprotein-Cholesterol, HDL-C: High Density Lipoprotein-Cholesterol.

Table 3, shows the results of the relative risks for different variables in two groups (group1: BLL > 40 μg/dl and group2: BLL ≤ 40 μg/dl).

**Table 3:** Risk estimate between workers with BLL >40 μg/dl and BLL ≤40 μg/dl

**Variables**	**Adjusted OR**	**CI% 95**	**P-value**
FBS ≥ 126 mg/dl	1.00	0.97–1.03	0.68
TG > 200 mg/dl	0.99	0.98–1.00	0.32
LDL-C > 130 mg/dl	0.99	0.98–1.00	0.17
HDL-C < 45 mg/dl	0.99	0.98–1.00	0.49
CHOL > 200 mg/dl	1.00	0.98-1.01	0.96
SBP ≥ 140 mmhg	1.01	0.99–1.04	0.12
DBP ≥ 90 mmhg	1.03	1.01–1.05	0.05

LDL-C: Low Density Lipoprotein-Cholesterol, HDL-C: High Density Lipoprotein-Cholesterol, CHOL: cholesterol, TG: Triglyceride, FBS: fasting blood sugar, SBP: systolic blood pressure, DBP: diastolic blood pressure, OR: odds ratio, CI %95: %95 confidence interval.

## Conclusions

Lead is a common environmental pollutant. Causes of environmental contamination include industrial use of lead, such as is found in plants that process lead-acid batteries or produce lead wire or pipes, and metal recycling and foundries.

This study showed that high diastolic blood pressure was more common in workers with BLL > 40 μg/dl than in workers with BLL ≤ 40 μg/dl (Odds ratio1.03 (95% CI: 1.01–1.05), p = 0.05). This finding persisted after adjustment was made for age, BMI, job experiences and smoking with p-value respectively 0.12, 0.80, 0.15, 0.54. This result was agreement with some previous studies [6,19-22], but some studies have shown that there were opposite effects [23-25].

Our study didn’t show statistically significant differences in total serum cholesterol and LDL-C level when comparing 2 groups (workers with BLL > 40 μg/dl and BLL ≤ 40 μg/dl). These results are consistent with previous study (34). But some studies have shown that high total serum cholesterol and LDL-C level to be more prevalent in the workers with high blood lead level than the workers with low blood lead level [6,19,20].

Studies in both humans and animals indicate that lipid metabolism is altered in chronic lead exposure [26,27]. The pathophysiological mechanisms involved in lead induced alterations are not completely understood.

We found no difference in the prevalence of hypertriglyceridemia when comparing workers with BLL ≤ 40 μg/dl and workers with BLL > 40 μg/dl. Our result was agreement with study by Oladipo Ademuyiwa in Nigeria [24]. But some studies have been shown high serum triglyceride levels to be more prevalent among workers with BLL > 40 μg/dl than the workers with BLL ≤ 40 μg/dl [23,25].

In our study, there was no difference in the prevalence of HDL-cholesterol level between workers with BLL > 40 μg/dl and Workers with BLL ≤ 40 μg/dl. So this result was in agreement with the results in previous study [23-25]. But Hanne Kirkby et all, found low level of HDL-C in workers with BLL > 40 μg/dl [21].

We didn’t find difference in the prevalence of hyperglycemia between workers with BLL ≤ 40 μg/dl and BLL > 40 μg/dl, so this result in agreement with the result in previous study [17,18].

But Bener et all found high fasting blood sugar was more prevalent among workers with BLL > 40 μg/dl than the workers with BLL ≤ 40 μg/dl [19].

On the basis of the study by our group and other, it is recommended that in every work place where lead exposure is mandatory, a lead clinic should be established. Trained health care personnel of the lead clinic should monitor intermittently (preferably every six months) the state of the blood pressure of each lead exposed worker. Upon discovering significant increases in blood pressure, especially in diastolic blood pressure a transfer from lead exposed area to non lead exposed area of at least one year should be recommended to the employer/management. This would perhaps reduce the possibilities of ill-effects of lead exposure that are expected upon the workers.

Attention to this environmental risk factor through health-protective public policies, workplace modifications, individual behavioral changes, and also scheduling for periodic examination regularly with focusing on ischemic heart disease risk factors specially diastolic blood pressure in lead exposed workers is likely to decrease the public health burden of cardiovascular disease.

## Abbreviations

BLL: Blood lead level; LDL-C: Low density lipoprotein- cholesterol; HDL-C: High density lipoprotein-cholesterol; CHOL: Cholesterol; TG: Triglyceride; FBS: Fasting blood sugar; SBP: Systolic blood pressure; DBP: Diastolic blood pressure; BMI: Body mass index; OR: Odds ratio; CI% 95: %95 confidence interval

## Competing interest

The authors declare that they have no competing interests.

## Authors’ contributions

MG conceived and designed the study and prepared the manuscript. KA performed the statistical analysis and interpretation of data. AS Performed the data collection. RK revising it critically for important intellectual content. MG performed final approval of the version to be published. All authors read and approved the final manuscript.
